# Evaluation of the impact of blast damage on tunnel surrounding rock induced by C-type cumulative tube cone angle

**DOI:** 10.1038/s41598-025-31460-w

**Published:** 2025-12-13

**Authors:** Jian Guo, Honglu Fei, Hainan Ji, Shijie Bao, Gang Hu, Yu Yan

**Affiliations:** 1 Institute of Blasting Technology, Liaoning Technical University, Fuxin, 123000 China; 2 School of Civil Engineering, Liaoning Technical University, Fuxin, 123000 China; 3 School of Mining Engineering, Heilongjiang University of Science and Technology, Harbin, 150022 China

**Keywords:** Cumulative blasting, Cumulative cone angle, Tunnel excavation, Smooth blasting, Crack propagation, Engineering, Natural hazards, Solid Earth sciences

## Abstract

Over-excavation and under-excavation are common issues in tunnel construction using the drilling and blasting method. In some cases, traditional blasting techniques may fail to achieve the desired results. This study systematically investigates the blasting-induced damage effects of C-type cumulative tubes with varying cone angles in different grades of tunnel surrounding rock, aiming to mitigate over-excavation and under-excavation issues while enhancing construction efficiency. First, the working principles of cumulative charges and their mechanisms in rock penetration and fragmentation were systematically analyzed based on the fundamental theory of cumulative blasting. Subsequently, numerical models of single-hole cumulative blasting were developed for both III-level and IV-level surrounding rock conditions to examine the influence of cumulative cone angles on rock crack propagation. Finally, field validation tests were conducted at a tunnel construction site in Chamdo, Tibet. Results showed that a 65° cone angle provided the best performance in III-level rock, while a 55° angle worked best in IV-level rock. Compared to traditional methods, the cumulative charge method increased contour hole spacing by 12 ~ 18% and reduced construction costs by 23.5%. It also cut over-excavation by 58.3% and improved half-hole preservation by 41.7%, ensuring better excavation profile integrity and effectively addressing excavation issues.

## Introduction

The drilling and blasting method has become the predominant technique for mountain tunnel excavation due to its advantages in cost-effectiveness, operational flexibility, and construction efficiency. However, its application presents a challenge in balancing the controlled blasting of rock mass within the designed excavation profile with the preservation of surrounding rock mass outside the profile. Currently, smooth blasting technology has been widely applied in the blasting excavation of mountain tunnels. In comparison to conventional blasting methods, smooth blasting demonstrates significant improvements in achieving precise contour shaping and offers substantial reductions in construction costs^1–3^. However, certain limitations remain in addressing varying grades of surrounding rock and fulfilling the demands of modern tunneling practices characterized by rapid excavation and precision construction. These limitations include insufficient spacing of peripheral blast holes in smooth blasting operations^4^, pronounced over-excavation and under-excavation phenomena^5^, and considerable disturbance to the preserved rock mass^6^. The conventional smooth blasting technique is no longer sufficiently capable of meeting the increasingly stringent demands of modern tunnel engineering, particularly in terms of rapid execution, precision, and minimal disturbance during construction.

In smooth blasting operations, such as slope blasting in open-pit mines, tunnel excavation and underground cavern development, it is required to keep the damage to the surrounding rock mass to the minimum extent. Over the past few decades, significant efforts have been dedicated to investigating the damage effects of blasting on rock masses, with a primary objective of minimizing the adverse impacts associated with blasting operations^7–12^. Practical experience from large-scale rock mass engineering excavation indicates that an appropriate excavation sequence and the application of contour blasting technology have a direct impact on construction quality, project progress, and economic efficiency^13–17^. To ensure the integrity and stability of the surrounding rock mass, the cumulative blasting excavation technique is extensively utilized in tunneling engineering applications.

The accurate prediction of rock mass damage caused by blasting operations is of critical significance in geotechnical engineering. In recent years, a large number of research achievements have emerged in the field of smooth blasting. Research conducted by Wang et al.^18^ and Xu et al.^19^ demonstrates that the slotted charge structure significantly enhances energy accumulation and propagation along the cumulative direction, while simultaneously reducing energy transfer and borehole wall pressure in non-cumulative directions. With respect to the cumulative charge structure itself, existing research has established that its material composition and structural parameters directly influence blasting effectiveness. Zhang et al.[^20^] systematically investigated key structural variables and concluded that an arc-shaped charge configuration achieves optimal blasting outcomes. Ding et al.^21^ found that double-slot charge designs exhibit better performance than triple-slot alternatives. Some scholars^22,23‚24^ experimentally discovered that crack-induced damage values along the cumulative direction of boreholes are substantially higher than those observed in non-cumulative directions. Furthermore, numerical simulations performed by several researchers^25–28‚29^ suggest that the eccentric placement of grooved pipes plays a decisive role in determining subsequent blasting results. The application of cumulative tube charges in perimeter holes within fault zones has effectively reduced over-break occurrences. In summary, Cumulative blasting technology utilizes precision-engineered lined devices to generate controlled tensile stress fields along predefined orientations in rock formations. This technique enables directional fracture initiation through concentrated energy projection, achieved by modifying explosive charge configurations to localize energy release along specified vectors. The methodology demonstrates three operational advantages: (1) directional energy focusing through geometric confinement of detonation waves, (2) significant reduction of collateral rock damage via minimized radial stress propagation, and (3) enhanced excavation precision through fracture path control. By optimizing the explosive energy distribution pattern, this technology effectively addresses critical engineering challenges including perimeter overbreak mitigation, underbreak prevention, and preservation of rock mass integrity, while maintaining high fracturing efficiency in the designated propagation plane^30–36‚37^.

**Fig. 1:**
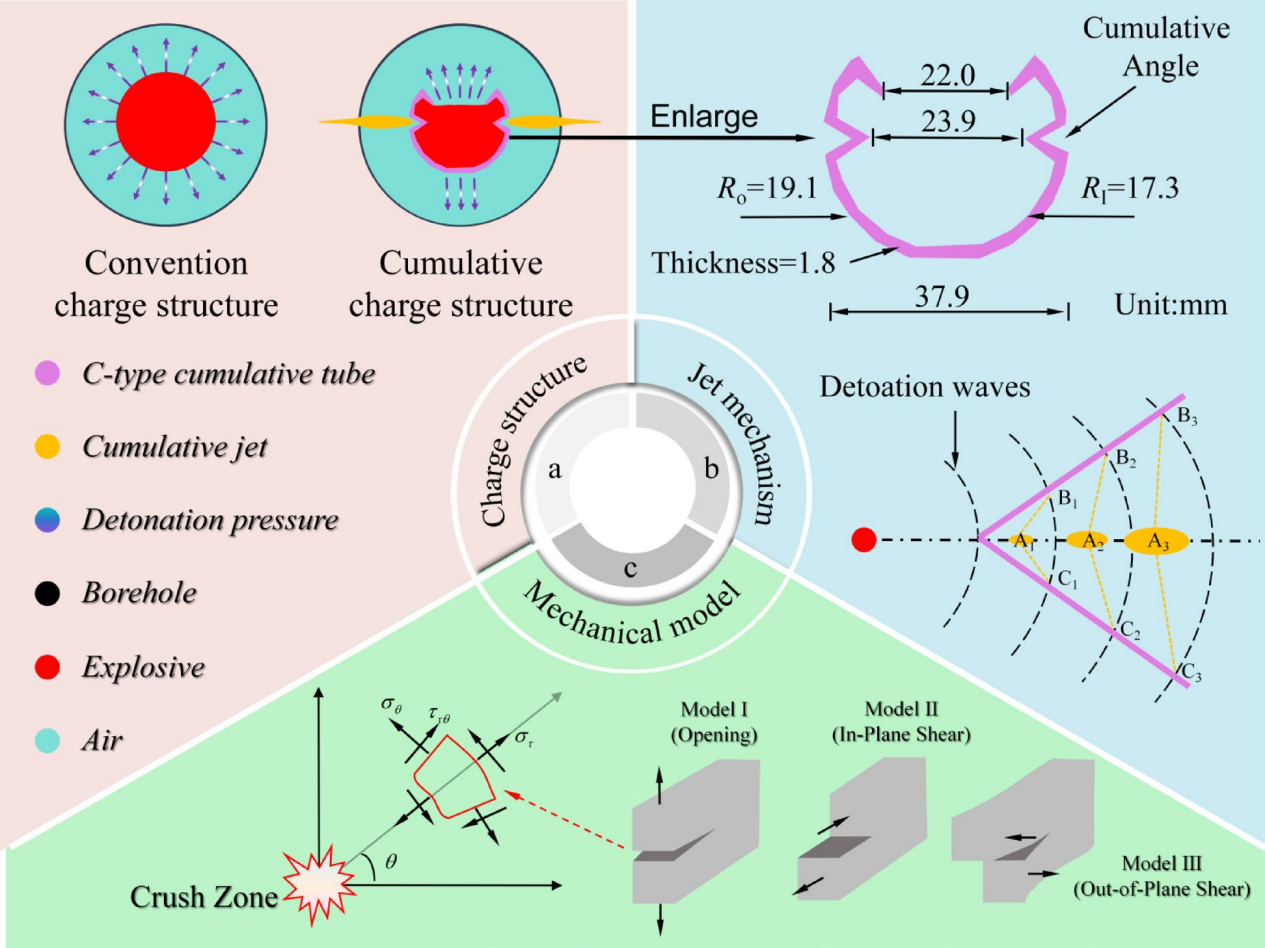
Schematic diagram of the cumulative charge structure.

However, in practical applications, a critical challenge remains in appropriately matching the angle of cumulative tubes with different surrounding rock grades, as this mismatch often prevents the attainment of optimal blasting performance. To overcome these shortcoming, This study employs an angle-adjustable C-type cumulative tube (see Fig. 1) to conduct an in-depth investigation into the rock crack propagation characteristics under different cone angle conditions. The numerical simulation models were established to comprehensively investigate rock mass fracture patterns in different surrounding rock conditions under blasting loads, while uncovering the intrinsic mechanisms governing rock failure with varied cumulative cone angles. Finally, the smooth blasting control methods for mountain tunnels under III-level and IV-level surrounding rock conditions were proposed and verified based on the field practice of cumulative blasting excavation in a tunnel located in Chamdo, Tibet. The research findings offer crucial guidance for subsequent blasting energy regulation and process parameter optimization in mountain tunnel engineering.

### Mechanism of rock fracture by C-type cumulative tube

The C-type cumulative tube (CCT) can effectively concentrate the energy produced during an explosion by optimizing the charge structure, thereby generating a high-velocity, high-temperature, and high-pressure jet. This jet is capable of penetrating the rock surface and initiating internal cracks within the rock mass. The blasting fracture mechanics model of the CCT structure is illustrated in Fig. 1.

When the explosive within the charge device is initiated, the specifically designed CCT structure (see Fig. 1b) effectively directs the energy released from the explosion along a predetermined propagation path. The explosive initiation generates a powerful detonation wave, which uniformly diffuses through the surrounding medium. Upon reaching the energy-gathering device, the detonation waves sequentially induce collision and compression at specific points—such as A_1_, A_2_, and A_3_—resulting in the formation of new curved surfaces, including but not limited to C_1_A_1_B_1_, C_2_A_2_B_2_, and C_3_A_3_B_3_. Furthermore, the explosive energy propagates radially along the borehole axis, generating a high-velocity jet. This jet exhibits substantial penetration capacity, thereby promoting the initiation and propagation of fractures in the designated direction of the charge structure. The stress *P*_1_ generated by the cumulative jet acting on the rock mass at the borehole location is^38^:

1 $$P_{1} = \frac{{\lambda R_{0} {\mathrm{cos}}\alpha }}{r(1 + k)}\rho_{0} D^{2}$$

where $\lambda$ is the pressure amplification coefficient; *R*_0_ is the equivalent radius of cumulative explosive (m); *r* is the borehole radius (m); *k* is the isotropy index of detonation products (usually taken as 3); $\rho_{0}$ is the density of explosives (kg/m^3^); *D* is the detonation velocity of explosives (m/s).

As indicated by Eq. 1, a reduction in the opening angle of the cumulative tubes results in an increase in the magnitude of stress exerted on the rock mass adjacent to the borehole along the cumulative direction. During the crack propagation process, the stress intensity factor *K*_1_ at the crack tip is:

2 $$K_{1} = P_{1} \cdot B\sqrt {\pi (r + L)} + \sigma_{\theta } \sqrt {\pi L}$$

where *K*_1_ is the stress intensity factor of the model I; *B* is the correction coefficient; *L* is the instantaneous expansion length of the radial crack (m); $\sigma_{\theta }$ is the tangential stress caused by blasting (MPa).

When the stress intensity factor (*K*_1_) exceeds the fracture toughness of the rock mass (*K*_IC_), crack propagation initiates and continues, with the propagation behavior governed by the following formula:

3 $$P_{1} > \frac{{K_{IC} - \sigma_{\theta } \sqrt {\pi L} }}{{B\sqrt {\pi (r + L)} }}$$

The guiding slit created by the cumulative jet penetration is substantially larger than the minor cracks within the crushing zone. Following the detonation, this slit facilitates the entry of a significant quantity of high-pressure detonation gas. According to the principle of energy conservation, the effectiveness of the detonation gas in the non-cumulative direction decreases accordingly. Consequently, the cumulative charge enhances crack propagation in the focusing direction while simultaneously inhibiting crack development in the non-focusing direction.

### Numerical simulation

Numerical simulation offers a cost-effective and efficient alternative to theoretical analysis and industrial experiments in the study of rock fragmentation process. To investigate the effects of different cone angles of shaped charges on blasting performance, a numerical model of single-hole shaped charge blasting was developed using the explicit dynamic analysis software LS-DYNA. LS-DYNA, a nonlinear explicit finite element analysis software capable of handling complex dynamic problems via Lagrangian, Smoothed Particle Hydrodynamics (SPH), Eulerian, and Arbitrary Lagrangian–Eulerian (ALE) solvers, has been extensively validated in applications such as rock fragmentation analysis, blast load modeling, and nonlinear transient dynamics.

**Fig. 2:**
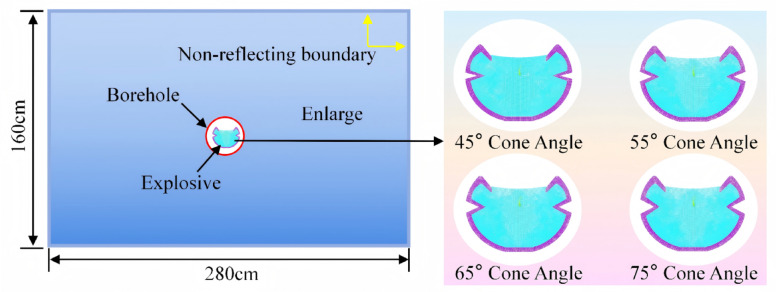
Schematic diagram of cumulative charge structure.

The commonly used cone angle α of the CCT ranges from 40° to 80°^39^. In this study, the cone angle is selected as a variable with values of 45°, 55°, 65°, and 75° (see Fig. 2), while other parameters—including the material of the shaped charge tube, wall thickness, opening configuration, and explosive type—are held constant. The detailed parameters of the CCT are presented in Fig. 1b. A total of eight numerical models of single-hole shaped charge blasting under grade III and IV surrounding rock conditions are established, corresponding to the different cone angles. Given that the length of the explosive is significantly greater than its diameter, the problem can be approximated under plane strain conditions^40^. The model dimensions are 280 × 160 cm, with a thickness of 1 cm. Furthermore, to explore the cumulative effect of the CCT structure, single-hole blasting models with conventional charge structures were established under grade III and IV surrounding rock conditions to facilitate comparative analysis. The borehole and explosive diameters are 5 cm and 3.2 cm, respectively. Finally, non-reflecting boundary conditions were applied to simulate an infinitely extended rock medium. Within the scope of this study, a fluid–solid coupling algorithm was employed.

### Material parameters

To thoroughly investigate the influence of different conical angles of the C-type cumulative tube on crack propagation behavior, this study employed the Holmquist-Johnson–Cook (HJC) constitutive model to characterize rock material properties. The model incorporates multiple key factors, including compaction and fragmentation effects, strain rate sensitivity, damage evolution, and confining pressure, thereby enabling a more accurate simulation of the deformation and failure behavior of rocks under impact loading^41^. The HJC model parameters (granite) are summarized in Table 1.

**Table 1:** Rock mass model parameters.

Materials	Density *ρ* (g/cm^3^)	Poisson ratio	Shear modulus *G* (GPa)	Compressive strength *f*_c_ (MPa)	Tensile strength *T* (MPa)
III-level	2.7	0.25	28	154	12.2
IV-level	2.6	0.26	24.17	119	8.2

Upon detonation of explosives within a borehole, an instantaneous chemical reaction takes place, transforming the explosive material from a condensed solid state into a high-temperature, high-pressure gaseous state. The Jones-Wilkins-Lee (JWL) equation of state (EOS), widely recognized for its applicability in high-energy combustion scenarios, is commonly employed in numerical simulations to predict a wide range of pressures associated with explosive events. In this study, the JWL EOS is utilized to characterize the explosive behavior, and the corresponding equation is expressed as follows^42^:

4 $$P_{0} = A\left( {1 - \frac{\omega }{{R_{1} V}}} \right)e^{{ - R_{1} V}} + B\left( {1 - \frac{\omega }{{R_{2} V}}} \right)e^{{ - R_{2} V}} + \frac{\omega E}{V}$$

where *A*, *B*, *R*_1_, *R*_2_, and $\omega$ are the material parameters, respectively; *V* is the ratio of the initial specific capacity of the explosive. *E* is the initial energy density of the explosive.

In accordance with the field conditions, the present study incorporates the explosion parameters proposed by^43^, as presented in Table 2.

**Table 2:** Parameters for the explosive material and JWL EOS.

Explosive type	*Ρ*(g/cm^3^)	*D(*cm/μs)	*P*cj(GPa)	*A*(GPa)	*B*(GPa)	*R*_1_	*R*_2_	*ω*	*E*(J/mol)	*V*_0_
Emulsion explosives	1.3	0.42	5.2	214	0.182	4.2	0.9	0.15	4.195	1

The cumulative tube is fabricated from PVC material. During the initial phase of the explosion, as pressure and temperature rise rapidly, the cumulative tube progressive deformation leading to eventual failure. To accurately capture this deformation and failure behavior, the *MAT_PLASTIC_KINEMATIC material model is employed in this study, with the relevant parameters summarized in Table 3.

**Table 3:** Material parameters of cumulative tube.

Density *ρ* (g/cm^3^)	Poisson ratio	Shear modulus *G* (GPa)	Elastic modulus *E* (MPa)	Compressive strength *f*_c_ (MPa)
1.43	0.36	1.1	3.59	61.7

### Model parameter validation

**Fig. 3:**
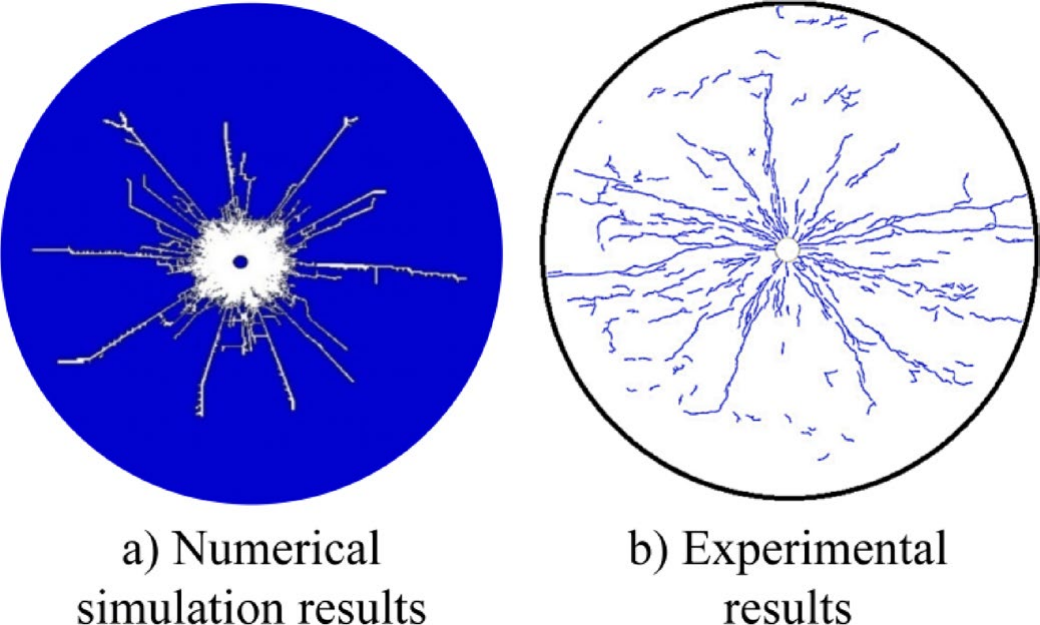
Comparison of experimental test and numerical simulation of blast crack extension pattern.

To verify the reliability of the model parameters, a single-hole blasting numerical model was established in LS-DYNA and compared with the laboratory test results of Banadaki et al^44^. The numerical simulation results of this paper are in good agreement with the experimental data (as shown in Fig. 3), indicating that the model can accurately simulate the rock damage process.

### Analysis of numerical simulation results

**Fig. 4:**
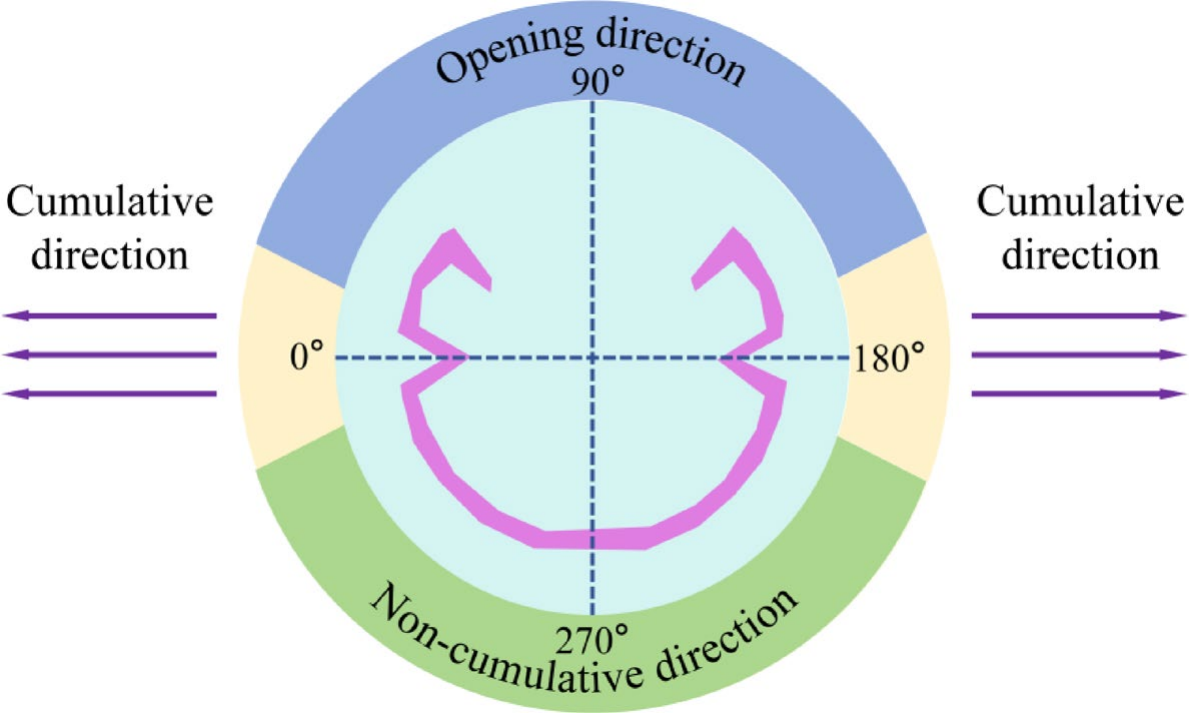
Schematic diagram of rock zone division.

The CCT exhibits notable energy regulation capabilities in three distinct directions: the cumulative direction, the opening direction, and the non-cumulative direction. The formation and high-velocity propagation characteristics of the jet are illustrated in Fig. 4. In the cumulative direction, the detonation wave front induces collapse of the focusing cover, leading to the generation of a high-speed jet that penetrates the surrounding rock and results in the formation of a rock pit. This initial rock fracture serves as a critical initiation point for guiding crack propagation along the intended cumulative direction in a stable and directional manner.

**Fig. 5:**
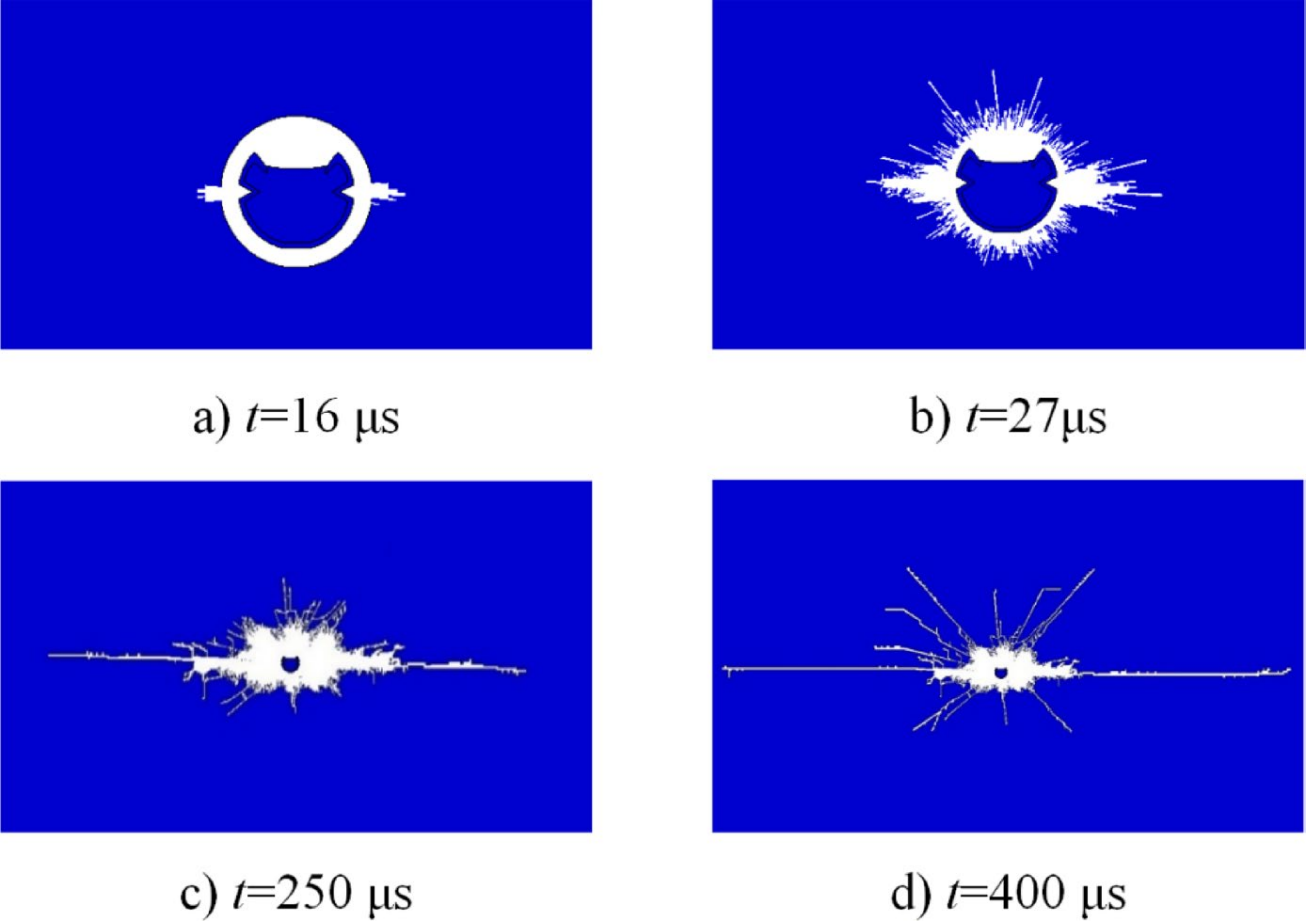
Crack propagation process of C-type cumulative tube blasting model (65°).

Given the observed similarities in crack propagation behavior across multiple models, the crack evolution process under III-level surrounding rock conditions with a 65° cone angle is presented in Fig. 5 as a representative example.

As illustrated in Fig. 5, a crack initiates and commences propagation along the cumulative direction (at *t* = 16 μs). At *t* = 27 μs, the crack propagation remains predominantly localized within the angular region of the cumulative cone, with an increased depth of penetration. By *t* = 250 μs, the primary crushing zones in both the cumulative and non-cumulative directions have become clearly defined, and the explosion-induced stress wave begins to interact with the rock mass, initiating fracture formation. At this stage, cracks propagate in multiple directions, with noticeable development also occurring in the non-cumulative direction. In the cumulative direction, the penetration effect of the focused jet results in significantly greater crack extension compared to the non-cumulative direction. This disparity in penetration and expansion becomes increasingly pronounced over time. When *t* reaches 400 μs, cracks in both directions are fully developed, and beyond this point, the cumulative jet no longer contributes to further crack initiation or rock penetration.

**Fig. 6:**
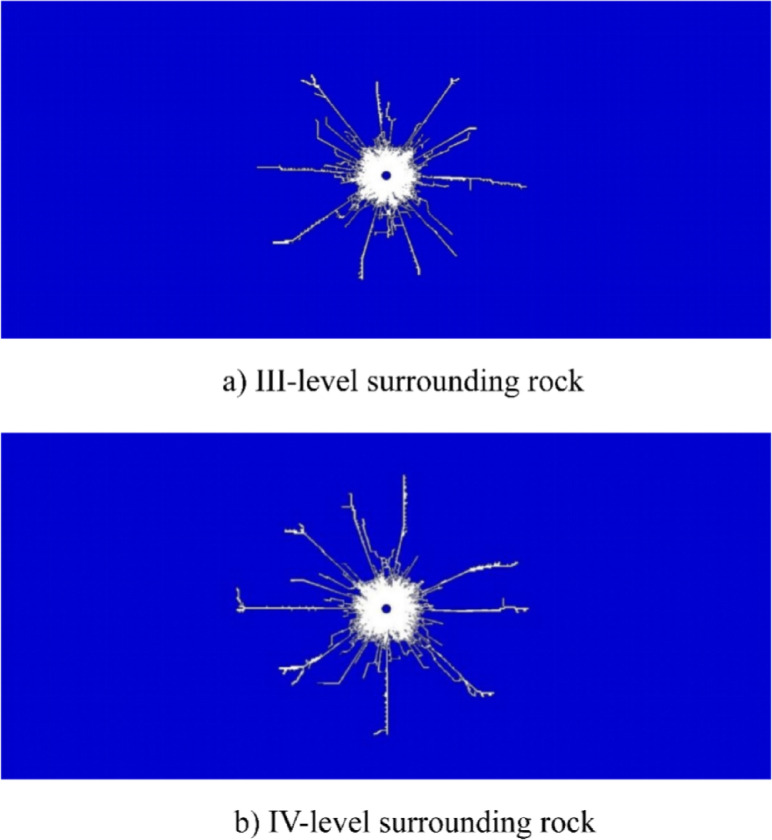
Simulation results of crack propagation in conventional blasting under different rock mass grades.

As illustrated in Fig. 6a, during the conventional blasting process, detonation products propagate outward in an approximately circular pattern, resulting in relatively uniform pressure distribution along the borehole wall. This uniform stress field contributes to the formation of cracks in the surrounding rock mass following fragmentation, exhibiting a radially expanding morphology. A significant portion of the explosive energy is dissipated in rock comminution, leading to an expanded crushed zone with a larger radius, while the fissure zone displays a concentrically radiating crack pattern. Such failure characteristics tend to induce extensive damage in the direction of the preserved rock mass, thereby causing pronounced overbreak. As depicted in Fig. 6b, compared to the blasting outcomes under III-level surrounding rock conditions, conventional blasting conducted under IV-level surrounding rock conditions results in a more extensive crushed zone and a denser network of fractures within the fissure zone.

### Simulation results of crack propagation in rock

**Fig. 7:**
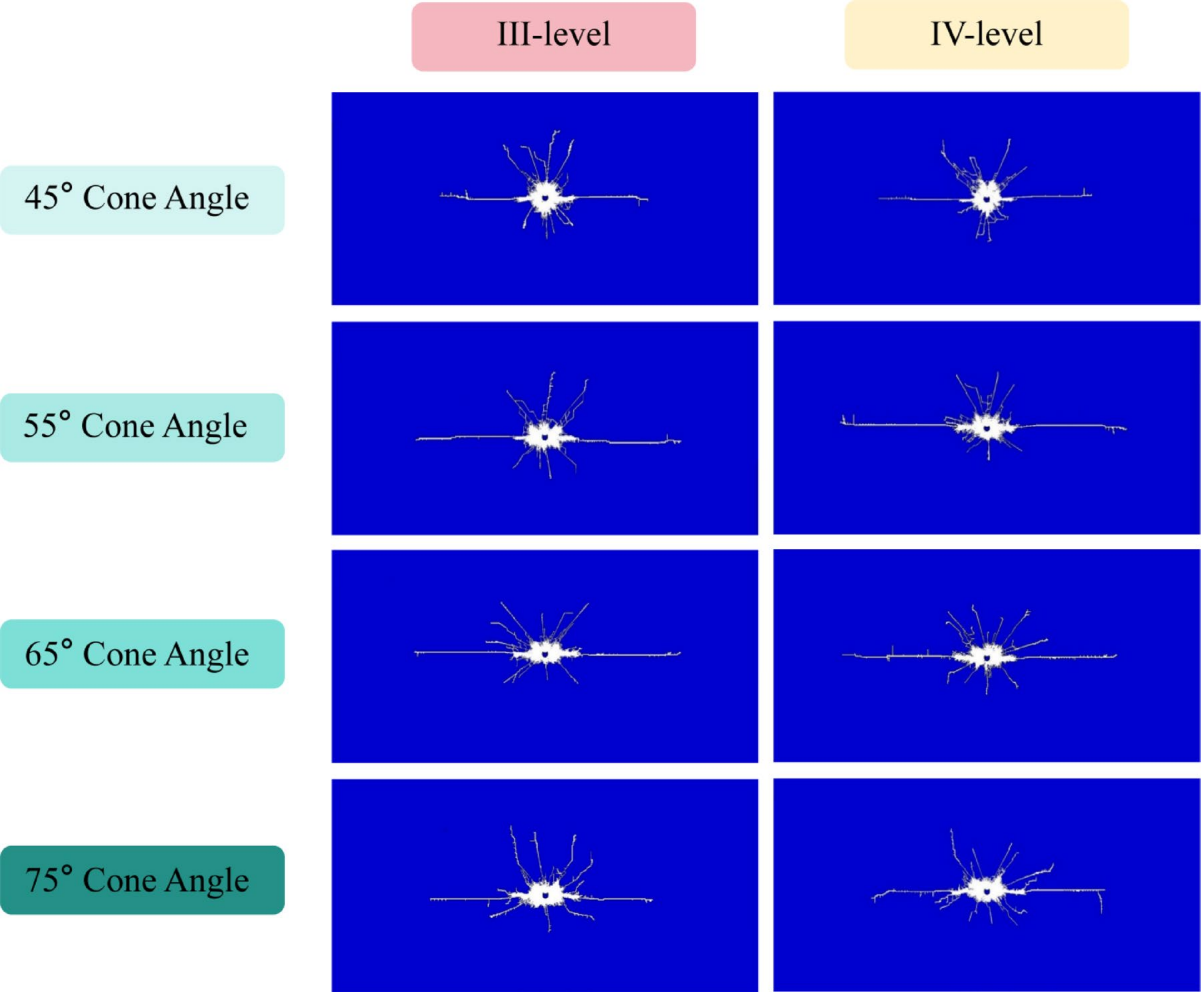
Simulation results of crack propagation under different surrounding rock conditions.

To systematically investigate the effect of cone angle on crack propagation behavior, four distinct CCT models with different angular configurations were established (see Fig. 2), and a comparative analysis was performed in relation to the conventional charge structure model (see Fig. 6). The numerical simulation results depicting rock crack propagation under different surrounding rock conditions are presented in Fig. 7.

The unique internal structural design of the CCT enables the focusing of explosive energy within the crushing zone. During the early stage of crushing zone formation, the cumulative jet initially penetrates the rock mass. This high-pressure jet exerts concentrated impact on the rock, inducing significant local stress concentration and rapidly generating initial cracks at the contact interface. These primary cracks act as structural weaknesses in the rock mass, thereby lowering its fracture initiation pressure (The findings are consistent with those reported by Peng et al. regarding rock failure mechanisms obtained through triaxial dynamic impact tests and uniaxial compression tests.^45,46^) and offering preferential pathways for the subsequent expansion of detonation gases (see Fig. 7).

The cumulative jet initiates directional cracks along a specific orientation, which subsequently facilitates further extension and propagation of these cracks through the action of explosive gases. The energy-focusing effect of the shaped charge groove generates a high-velocity jet, substantially enhancing the efficiency of rock-breaking energy utilization in the designated direction. This improved efficiency ensures effective energy deployment, resulting in a relatively smaller crushing zone and reduced energy consumption, thereby promoting more pronounced crack propagation in that direction.

In the non-cumulative direction, the blasting process follows a mechanism similar to that of conventional charge configurations: detonation energy initially impacts the outer shell of the CCT. Upon shell rupture, the resulting shock wave propagates to the borehole wall and the surrounding rock mass. Throughout this process, the PVC tube serves as an energy barrier, effectively attenuating energy transmission and thereby minimizing the impact on the retained rock mass behind the blast zone. This attenuation leads to a reduction in both the extent of the crushed zone and the degree of crack propagation in this direction.

**Fig. 8:**
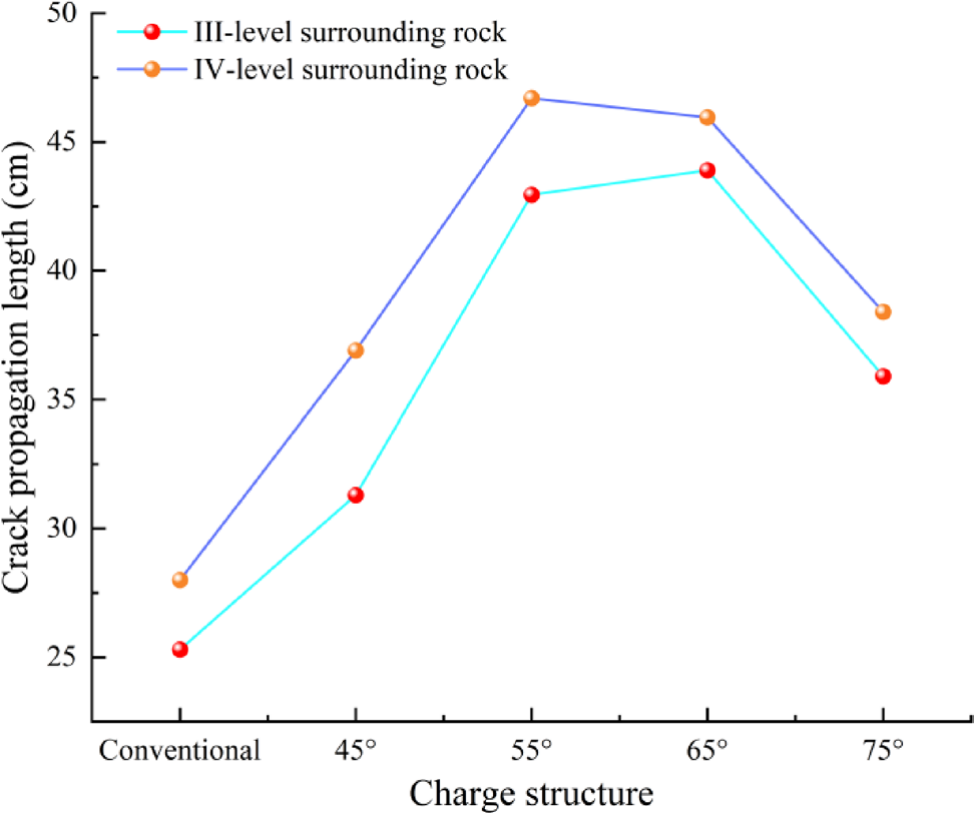
Crack propagation lengths along the cumulative direction under different cone angles.

During the formation of the fragmentation zone and crack zone, both the fragmentation zone radius and crack propagation length exhibit significantly greater values in the direction aligned with the shaped charge’s conical angle compared to other directions. Under IV-level surrounding rock conditions, the blasting performance—whether employing conventional charge or CCT—demonstrates comparable characteristics to those observed under III-level surrounding rock conditions. The crack lengths measured along the cumulative jet direction for each model are presented in Fig. 8.

As illustrated in Fig. 8, when all other conditions remain constant, the length of radial cracks along the cumulative direction initially increases and subsequently decreases with the increment of the conical angle. The simulation findings indicate that an excessively small conical angle results in excessive energy concentration within a limited area, thereby reducing energy utilization efficiency and hindering effective crack propagation outward. Conversely, as the conical angle increases, the jet coverage expands, leading to a more uniform distribution of energy over a broader region, which facilitates crack initiation and propagation across a wider spatial range. However, when the conical angle becomes excessively large, the energy disperses too widely, causing the crack extension length to reach a maximum and then decline.

In the context of III-level surrounding rock conditions, the maximum radial crack length is observed at a conical angle of 65° (III-65°). At this point, the maximum crack length has significantly increased by 73.5% compared to the crack length under conventional charge conditions. Similarly, under IV-level surrounding rock conditions, the radial crack length exhibits an analogous trend of initial increase. Compared to blasting in III-level surrounding rock, the optimal conical angle for achieving the longest radial crack length in IV-level surrounding rock is determined to be 55° (IV-55°). At this point, the maximum crack length has significantly increased by 66.8% compared to the crack length under conventional charge conditions.

**Fig. 9:**
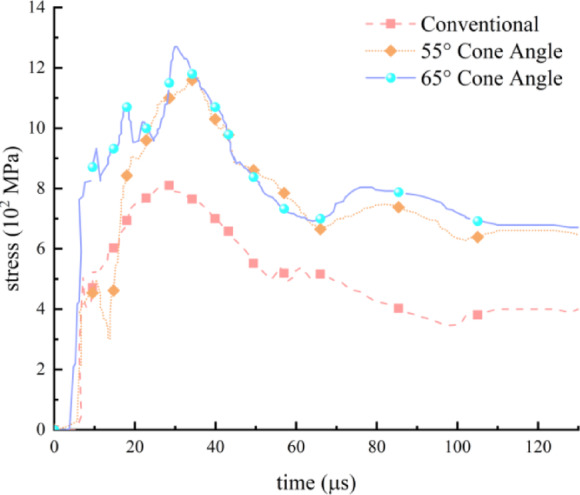
Stress-time variation curve of the unit element on the borehole wall.

Noteworthy, under two different surrounding rock conditions, the crack propagation effect of the cumulative cone angle within the range of 55° to 65° is superior to that of other angles. To thoroughly investigate the stress propagation characteristics along the cumulative cone angle direction, this study employs two cumulative charge models with conical angles of 55° and 65°, as well as a conventional charge model for comparative analysis. A representative element located on the borehole wall along the direction of the cumulative groove is selected for each model to plot the corresponding stress-time variation curve, as illustrated in Fig. 9.

As illustrated in Fig. 9, the stress attains its maximum value at approximately *t* = 28 μs, reaching a peak of 0.81 GPa, followed by a decline to 0.432 GPa. After this point, the stress exhibits a fluctuating yet decreasing trend over time. In the case of a 55° cumulative cone angle, the stress reaches a peak at around *t* = 27 μs, with a magnitude of approximately 1.16 GPa, followed by a sharp decrease to 0.738 GPa. Subsequently, the stress undergoes minimal fluctuations and gradually diminishes. For a 65° cumulative cone angle, the stress peaks at approximately *t* = 26 μs, with a maximum value of 1.27 GPa, followed by a rapid drop to 0.804 GPa. Thereafter, the stress remains relatively stable with only minor variations while continuing to decrease gradually.

As illustrated in Fig. 9, the CCT demonstrates a significantly enhanced capability in energy concentration compared to the conventional charge structure. Specifically, when the cumulative angles are set at 55° and 65°, the peak stress generated by the CCT reaches 1.43 and 1.57 times that of the traditional charge, respectively. These results provide strong evidence that the CCT is capable of forming a cumulative jet, thereby extending the duration of explosive energy penetration into the rock mass and ultimately achieving superior fracturing performance.

### Single-hole cumulative blasting comparison test

To verify the reliability of the numerical simulation results, C-type cumulative tubes with cone angles of 55° and 65° were selected for single-hole blasting experiments under Ⅲ-level and IV-level rock mass conditions. During the experiments, the NM-4A non-metallic ultrasonic detector and analyzer were utilized to detect and analyze the rock mass damage after blasting. These experimental data were used to further validate the optimal cone angle of the C-type cumulative tubes under different rock mass conditions.

**Fig. 10:**
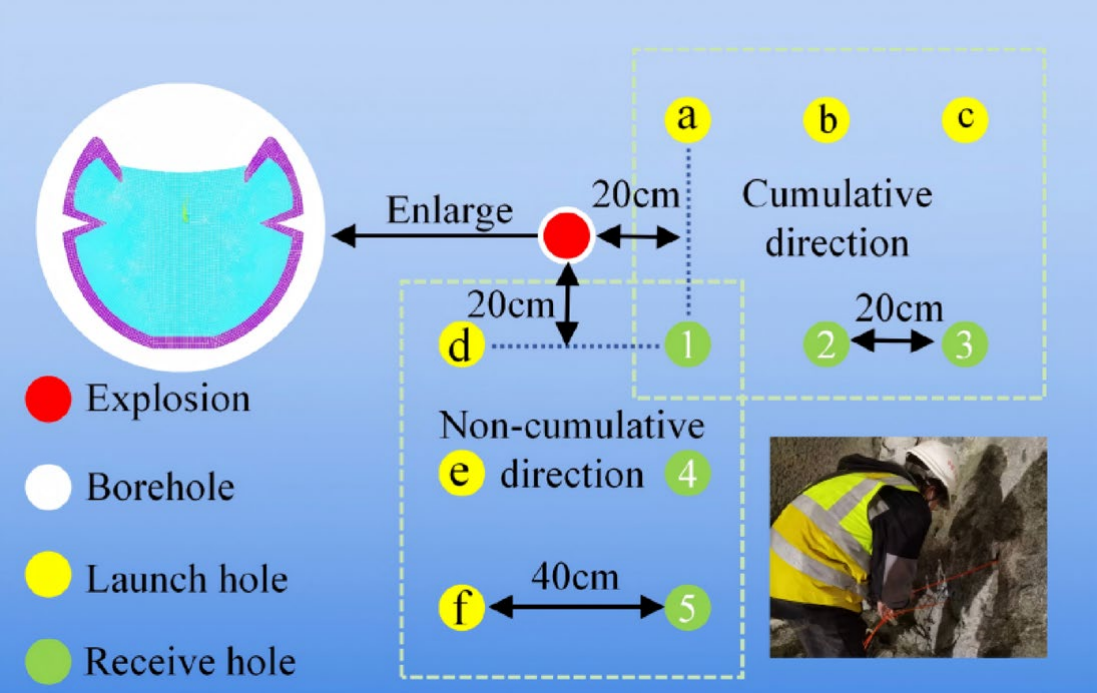
Schematic diagram of single hole blasting test drilling.

On the construction site, 2# rock emulsion explosives were used, with a charge diameter of 32 mm and a length of 10 cm (Located at the middle position of the borehole). The borehole had a diameter of 50 mm and a depth of 1.5 m. The borehole was plugged with mud from the bottom of the tunnel, with a plug length of 20 cm. Apart from the plug, the air medium in the borehole was filled with water bags to convert the air medium charge into a water medium charge. The spacing between the acoustic receiver hole and the transmitter hole was 40 cm, while the spacing between the transmitter holes was 20 cm (see Fig. 10). Inclined drilling was adopted to facilitate water injection into the test hole and to ensure the accuracy and reliability of acoustic detection.

### Acoustic data collection

This test was conducted in the form of small-scale blasting experiments, aiming to accurately reflect the characteristics of acoustic wave propagation in rock masses under blasting conditions. Based on different rock mass grades, three sets of single-hole blasting tests were carried out, with charging methods including conventional charge, 55° and 65° C-type cumulative tubes. During the testing process, special attention was paid to maintaining the horizontal alignment of the transmitter and receiver probes to ensure the accuracy of the test results.

An inter-hole testing method was used, with acoustic wave tests conducted after drilling in the blasting area to fully understand the acoustic wave propagation in the rock mass. A total of six profile lines were set up for detection, with the cumulative directions numbered as a-1, b-2, and c-3, and the rock mass retention directions numbered as d-1, e-4, and f-5, ensuring comprehensive coverage of the testing area.

In the acoustic wave testing process, to fully detect the propagation of cracks within the rock mass, the water surface at the hole opening was used as the coordinate origin, and the probe was slowly moved upward from the bottom of the hole, with tests conducted every 5 cm until the transmitter and receiver transducers were pulled out of the water level in the hole. To ensure the accuracy of the experimental data and the reproducibility of subsequent experiments, detailed records of the test data were made after each test.

## Results analysis and discussion

Rock masses typically harbor a multitude of micro-cracks, pores, and other initial defects that are randomly distributed throughout their interior. These defects are collectively termed micro-defects. When subjected to explosive stress waves, these micro-defects can expand and interconnect, thereby initiating the cracking or fracturing of the rock mass. The presence and propagation of both micro-cracks and macroscopic fractures not only lengthen the propagation path of acoustic waves through the rock mass but also cause a significant reduction in their propagation speed. Furthermore, the attenuation of wave velocity and amplitude is closely correlated with the total quantity and width of the fractures. Consequently, by monitoring changes in acoustic wave velocity, one can conveniently and effectively evaluate the development of micro-cracks within the rock.

Zhu et al.^47^ established the relationship between the degree of rock mass damage *D*_s_, the rock mass integrity coefficient *K*_v_, and the variation rate of acoustic wave velocity, *η*.

5 $$D_{s} = 1 - K_{v} = 1 - (1 - \eta )^{2} = 1 - \left( {1 - \frac{{v_{h} }}{{v_{p} }}} \right)^{2}$$

where *v*_h_ is the acoustic wave velocity of the rock mass after blasting (km/s); *v*_p_ is the acoustic wave velocity of the rock mass before blasting, (km/s).

**Fig. 11:**
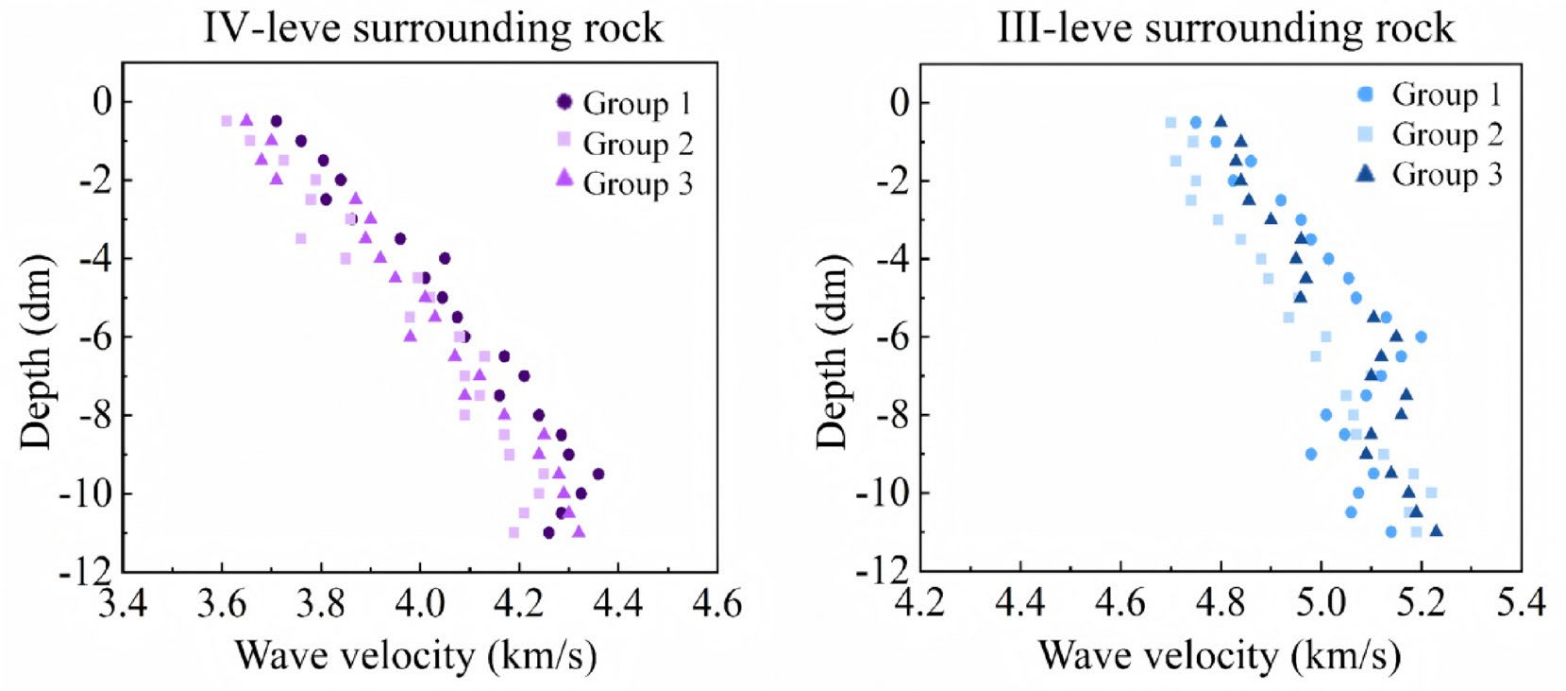
Sound wave propagation velocity in rock before explosion.

In this tunnel test, the rock mass at the test site is classified as III-level and IV-level surrounding rock, which exhibits good integrity. Therefore, the variation rate of acoustic wave velocity, *η* = 10%, was chosen as the threshold for rock mass damage. Accordingly, the degree of rock mass damage corresponding to this threshold is 0.19. Before the single-hole experimental blasting, we collected acoustic wave data at the original surrounding rock face to facilitate comparative analysis with the data obtained after blasting. Fig. 11 presents the results of acoustic wave data detection in the rock mass before blasting under III-level and IV-level surrounding rock conditions.

As shown in Fig. 11, under III-level surrounding rock conditions, the acoustic wave propagation velocity inside the borehole before blasting mostly ranges from 4750 to 5230 m/s. The wave velocity data before blasting in III-level surrounding rock indicates that the acoustic wave propagation velocity is higher near the bottom of the borehole. This suggests that the rock mass in this area has fewer internal pores and is more intact compared to the rock mass near the borehole opening. As the transducer is slowly moved upward from the bottom of the borehole, the acoustic wave propagation velocity gradually decreases. This indicates that the previous blasting cycle caused significant damage to the rock mass, with the degree of damage increasing as it approaches the free face. The borehole opening is located within the loosened zone of the surrounding rock formed by tunnel excavation, leading to more pronounced rock fractures near the borehole opening, which in turn significantly reduces the acoustic wave propagation velocity in this area. The trend of acoustic wave changes in IV-level surrounding rock is consistent with that in III-level surrounding rock. Under IV-level surrounding rock conditions, the maximum acoustic wave propagation velocity in the rock mass is 4320 m/s, and the minimum is 3610 m/s.

**Fig. 12:**
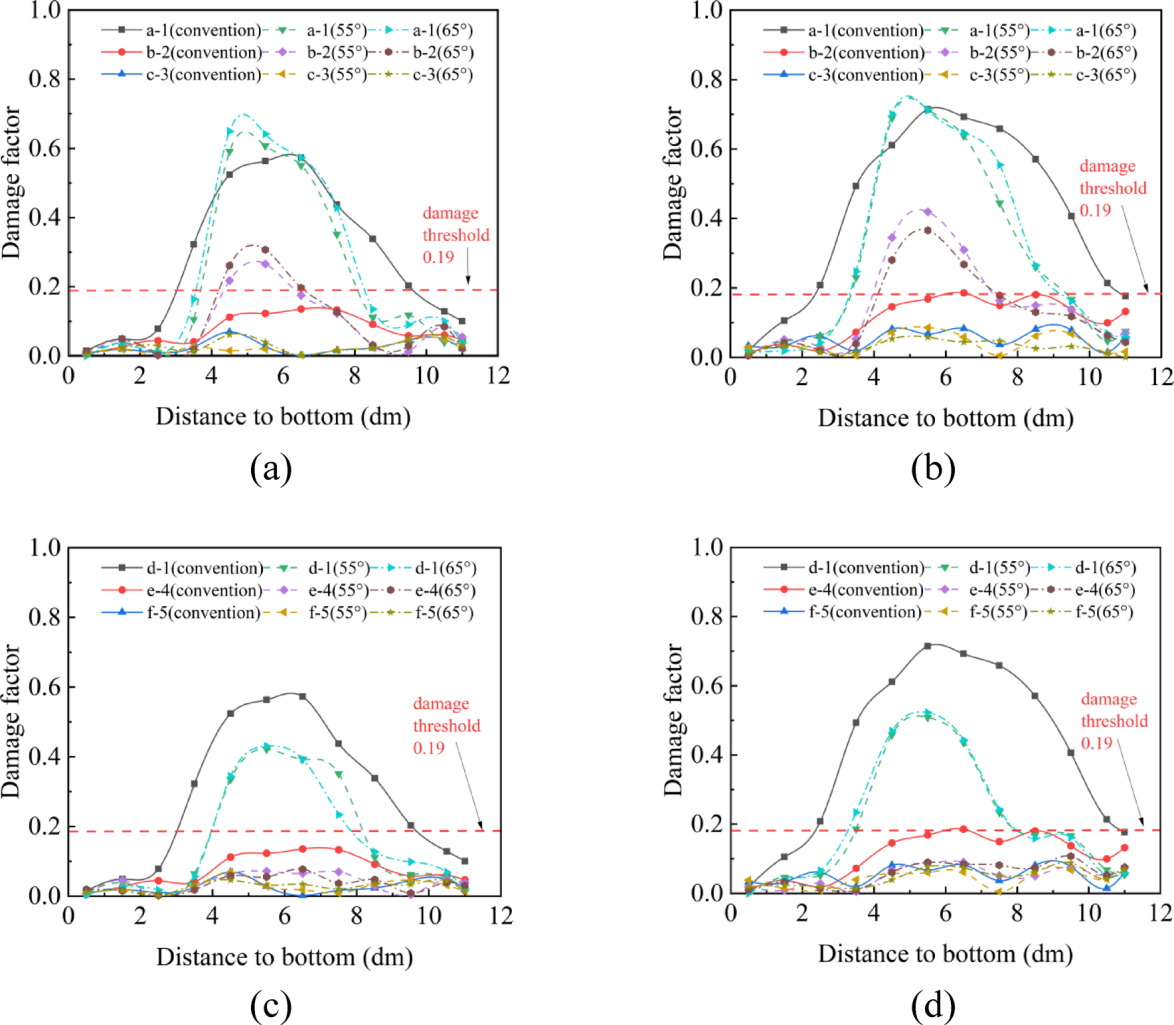
Rock mass damage data after blasting under different surrounding rock conditions (**a**) Cumulative direction of III-level surrounding rock; (**b**) Cumulative direction of IV-level surrounding rock; (**c**) Non-cumulative direction of III-level surrounding rock; (**d**) Non-cumulative direction of IV-level surrounding rock.

As can be seen from Fig. 12, the damage to the rock mass after blasting is mainly concentrated in the middle position of the borehole. In the direction of cumulative, compared with the conventional charge structure, the rock mass damage caused by 55° and 65° cone angles cumulative blasting at the b-2 detection hole is significantly increased. Under the condition of the conventional charge structure, the damage degree of the III-level surrounding rock at the b-2 detection hole is 0.13 (Fig. 12a), and the damage degree of the IV-level surrounding rock is 0.18 (Fig. 12b), both of which are below the damage threshold. This indicates that the rock mass damage range after the detonation of explosives is less than 40 cm. When using the C-type cumulative tube, the damage degree of the III-level and IV-level surrounding rocks at the b-2 detection hole is significantly increased. Under the condition of the III-level surrounding rock, compared with the conventional charge method, the damage at the b-2 detection hole using the 65° C-type cumulative tube is increased by 127%. Moreover, compared with the 55° conical angle, the damage degree is also increased by 15.4% (Fig. 12a). Under the condition of the fourth-grade surrounding rock, compared with the conventional charge method, the damage at the b-2 detection hole using the 55° C-type energy-concentrating tube is increased by 125%. Moreover, compared with the 65° conical angle, the damage degree is also increased by 14.6% (Fig. 12b).

In the non-cumulative direction (the direction of the reserved rock mass), regardless of whether it is a conventional charge structure or an cumulative charge structure, the damage at the b-2 detection hole does not exceed the threshold (Fig. 12c, d). However, compared with the conventional charge method, the damage degree at the d-1 detection hole is significantly reduced. Under the condition of the III-level surrounding rock, the rock mass damage degrees at this position for using the 55° and 65° conical angles are reduced by 20.5% and 23.6%, respectively (Fig. 12c). Under the condition of the IV-level surrounding rock, the rock mass damage degrees at this position for the 55° and 65° conical angles are reduced by 27.6% and 24.1%, respectively (Fig. 12d).

The above results show that the cumulative blasting using the C-type cumulative tube in the III-level surrounding rock condition can significantly increase the crack propagation degree in the cumulative direction, and the blasting effect of the 65° conical angle is better than that of the 55° conical angle. Under the IV-level surrounding rock condition, the blasting effect of the 55° conical angle is better than that of the 65° conical angle. In the direction of the reserved rock mass, the damage range of the conventional charge structure is greater than that of the cumulative charge structure. Therefore, matching the optimal energy-concentrating cone angle for different surrounding rock grades can not only reduce the damage to the reserved rock mass but also increase the borehole spacing and reduce the total amount of explosives used.

### Engineering application

The blasting test was conducted in a high-altitude, deep-valley region within the Three Rivers Basin in southeastern Tibet. The test site consisted of a double-tube separated extra-long tunnel with an overall length of approximately 5000 m. The main rock stratum of the tunnel body is granite.

The main tunnel, a double-track tunnel with III-level surrounding rock, is only slightly influenced by geological structures. Joint development is not evident, and no weak planes or interlayers are observed. The full-face excavation method is employed during construction. The cross-sectional area of the tunnel is 108.32 m^2^, with an excavation advance of 3.0 m per cycle. A single-level wedge-shaped cut technique is applied, involving a total of 196 boreholes. The total mass of explosive charge used per cycle is 417 kg, and the specific consumption of explosive of 1.28 kg/m^3^.

**Fig. 13:**
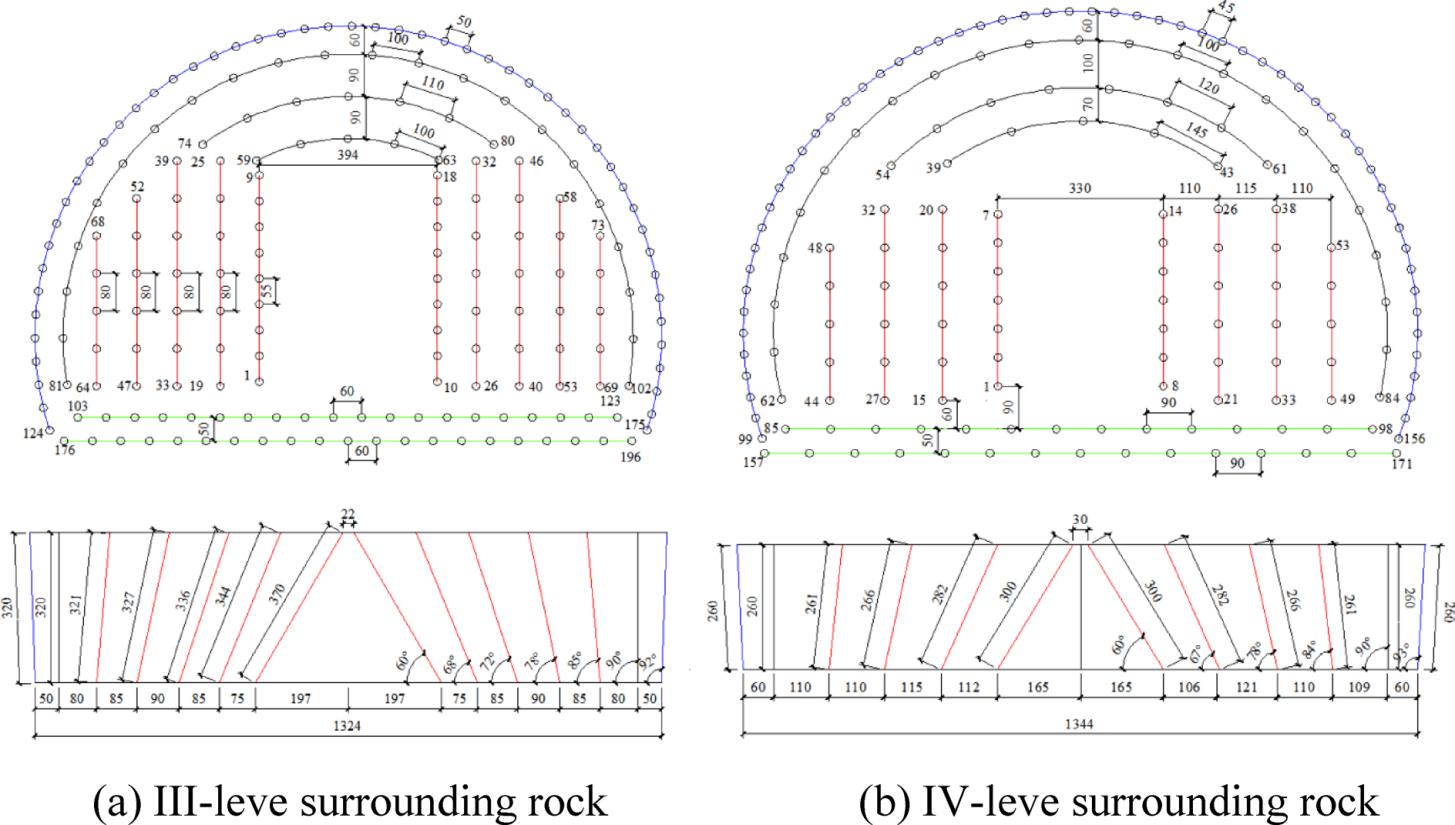
Full-section construction blasting design drawing of different surrounding rock (conventional design).

In addition, the surrounding rock in certain sections of the tunnel is classified as IV-level, which corresponds to hard rock. The rock mass is significantly affected by geological structures and exhibits well-developed jointing, along with a limited number of weak planes and through-going slightly open joints. The rock is predominantly slightly weathered, possessing strong self-stabilizing capacity. The cross-sectional area of the tunnel is 104.5 m^2^. During excavation, an advance length of 2.4 m is adopted using a single-level wedge cut blasting technique. A total of 171 boreholes are arranged, with a cumulative charge of 268.55 kg, and the specific consumption of explosive of 1.07 kg/m^3^. The blasting design for the full-face excavation of surrounding rock is illustrated in Fig. 13, and the corresponding technical parameters are summarized in Table 4.

**Table 4:** Design parameter table for full-section blasting of different surrounding rock (conventional design).

Rock mass classification	Name	Blasting number	Borehole depth(m)	Borehole spacing(m)	Delay interval(ms)
III-level	Cut hole	1–18	3.7	0.55	0
	Auxiliary hole	19–32	3.44	0.8	100
		33–46	3.36	0.8	200
		47–58	3.27	0.8	300
		59–63	3.27	1	300
		64–73	3.21	0.8	400
		74–80	3.21	1.1	400
		81–102	3.2	1	500
		103–123	3.2	0.6	500
	Contour hole	124–175	3.2	0.5	600
	Floor hole	176–196	3.2	0.6	700
IV-level	Cut hole	1–14	3	0.6	0
	Auxiliary hole	15–26	2.82	0.8	100
		27–38	2.66	0.8	200
		39–43	2.66	1.45	200
		44–53	2.61	0.8	300
		54–61	2.61	1.2	300
		62–84	2.6	1	400
		85–98	2.6	0.9	500
	Contour hole	99–156	2.6	0.45	500
	Floor hole	157–171	2.6	0.9	600

### Optimization and field application of contour hole blasting design

**Fig. 14:**
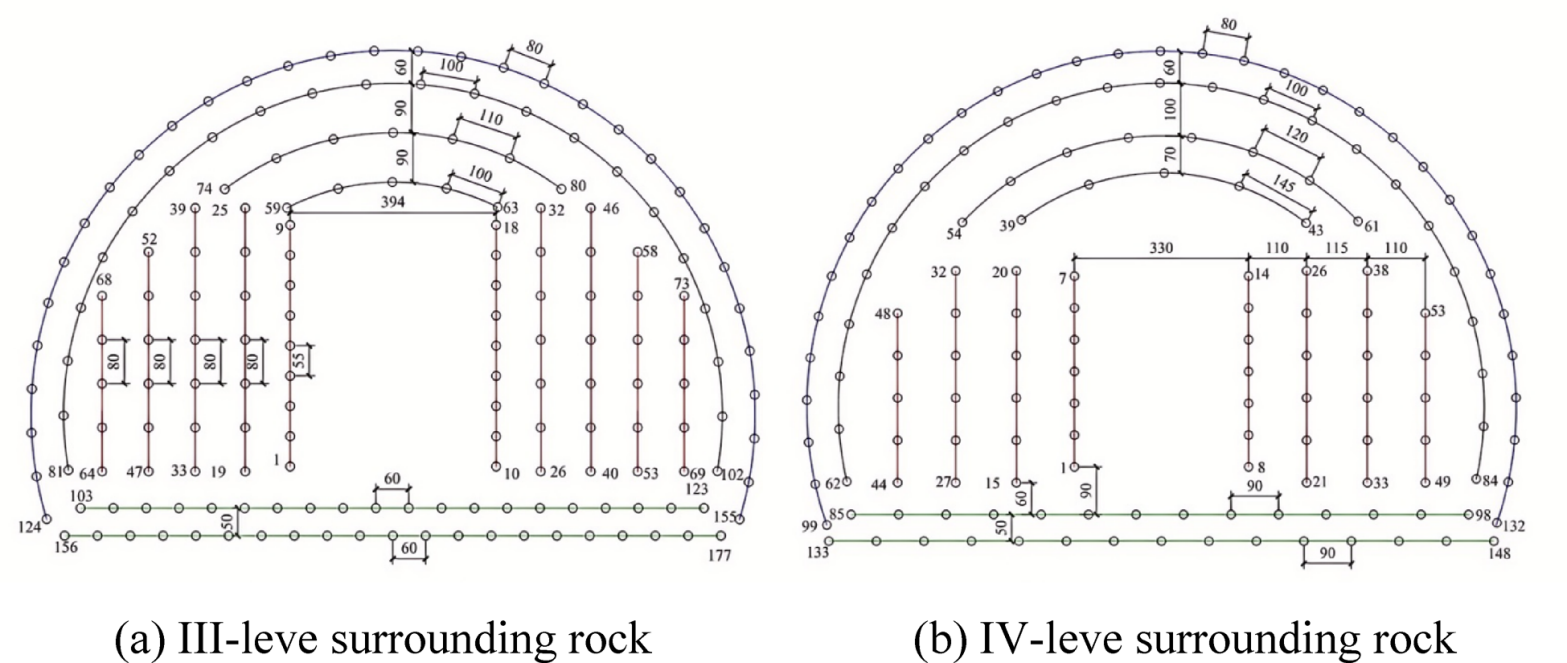
Full-section optimized construction blasting design drawing of different surrounding rock (optimum design).

In the conventional blasting design, the spacing between contour holes was established at 50 cm, while the burden distance of the smooth blasting range was set to 60 cm. For the cumulative blasting design, the structure of loading charge adopts CCT, and the spacing between contour holes was increased to 80 cm (Other parameters remained invariant while exclusively modifying the contour borehole spacing configurations). Concurrently, minor modifications were made to the arrangement of the remaining boreholes. The detailed layout scheme of the boreholes is illustrated in Fig. 14.

Following the optimization, the number of contour holes was reduced by 19 under III-level rock conditions and by 25 under IV-level rock conditions. This enhancement has substantially decreased drilling time and improved the overall efficiency of each tunnel excavation cycle.In the original charge structure, enhanced charge structure are employed at the bottom of the boreholes. The cumulative charge structure, with a total length of 2.8 m, adopts the same loading methodology as conventional configurations.

**Fig. 15:**
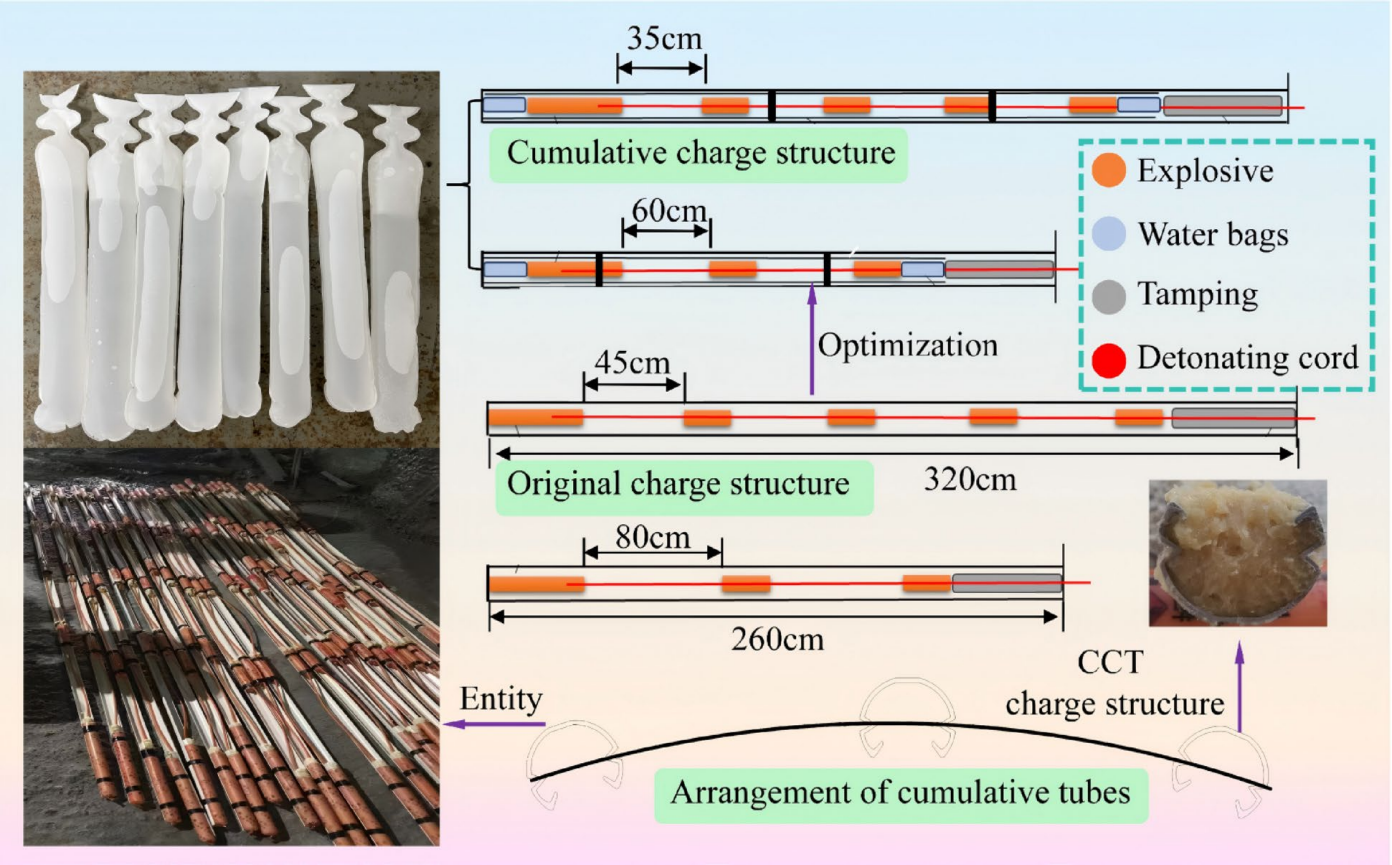
Schematic diagram of charge structures.

By contrast , water bags are incorporated at both the bottom and the top of the borehole. The energy storage capacity of water, combined with the water wedge effect, contributes to enhanced energy utilization efficiency of explosives and effective suppression of dust generation. The water bags utilized at the site have a length of 20 cm, a diameter of 27 mm, and a thickness of 0.8 mm. Prior to sealing the filling aperture, it is recommended to evacuate the internal air to the greatest extent practicable. Furthermore, it is essential to ensure that the orientation of the energy concentration grooves in the CCT aligns precisely with the tunnel excavation profile. Detailed placement specifications are illustrated in Fig. 15. The project employs 2# rock emulsion explosive, which is initiated using industrial electronic detonators.

The energy storage capacity of water, combined with the water wedge effect, contributes to enhanced energy utilization efficiency of explosives and effective suppression of dust generation. The water bags utilized at the site have a length of 20 cm, a diameter of 27 mm, and a thickness of 0.8 mm. Prior to sealing the filling aperture, it is recommended to evacuate the internal air to the greatest extent practicable.Furthermore, it is essential to ensure that the orientation of the energy concentration grooves in the CCT aligns precisely with the tunnel excavation profile. Detailed placement specifications are illustrated in Fig. 15. The project employs 2# rock emulsion explosive, which is initiated using industrial electronic detonators.

**Fig. 16:**
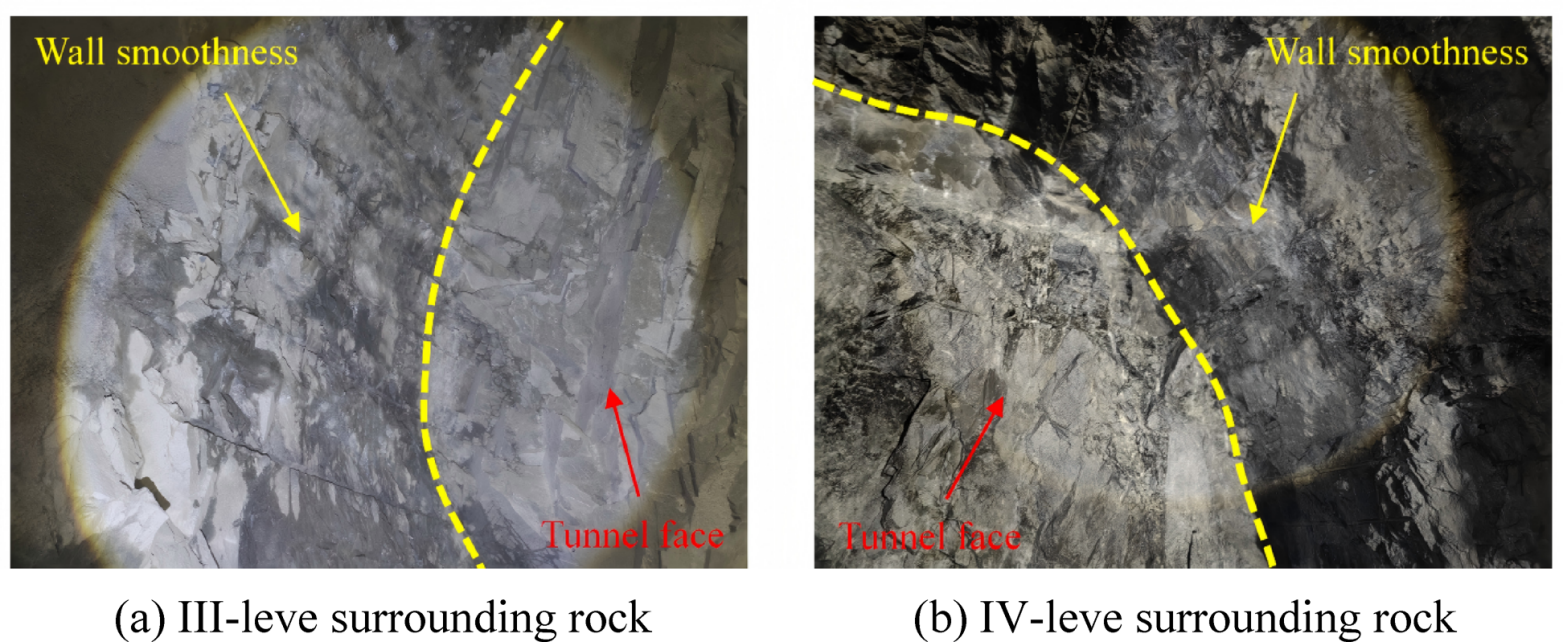
Tunnel profile surface after blasting.

In practical construction, for III-level surrounding rock condition, a CCT with a cone angle of 65° is employed, whereas for IV-level surrounding rock, a similar device with a cone angle of 55° is utilized. The reverse initiation technique is applied during the blasting process. To evaluate the effectiveness of the cumulative water pressure smooth blasting, parameters such as over-excavation of the tunnel profile surface and the half-hole residual rate are systematically monitored and analyzed. Detailed results of this evaluation are presented in Table 5. Upon completion of the blasting operations, the resulting tunnel profile surface is illustrated in Fig. 16.

**Table 5:** Comparison of blasting effects.

Classification	Grade	Over-excavation area(m^2^)	Maximum over-excavation (m)	Half-hole residual rate(%)
Conventional	III-level	5.969	0.564	51
	IV-level	10.027	0.974	76
Cumulative	III-level	4.614	0.386	75
	IV-level	8.244	0.581	93

As presented in Table 5, under III-level rock mass conditions, the application of 65° cone-angle cumulative blasting results in a 22.7% reduction in over-excavation area per cycle compared to conventional blasting methods. Additionally, the maximum over-excavation value decreases by 46.1%, while the half-hole retention rate increases by 24%. In IV-level rock mass conditions, the use of 55° cone-angle shaped charge blasting leads to a 17.8% decrease in over-excavation area per cycle, a 40% reduction in the maximum over-excavation value, and a 17% improvement in half-hole retention rate compared to conventional techniques. Furthermore, the implementation of CCT yields significantly more visible blast hole traces than the original design, with a smoother post-blast rock wall surface. This approach effectively minimizes disturbances and damage to the surrounding rock in the direction of the preserved rock mass, thereby contributing to the overall integrity of the excavation profile.

## Discussion

The findings of this study provide valuable insights into the optimization of blasting techniques in tunnel construction, particularly in the context of using C-type cumulative tubes (CCT) with varying cone angles. The numerical simulations and field tests conducted in this research demonstrate that the cone angle of the CCT plays a crucial role in determining the efficiency of rock fragmentation and the extent of damage to the surrounding rock mass. Specifically, a 65° cone angle was found to be optimal for III-level surrounding rock, while a 55° cone angle performed best in IV-level surrounding rock. These results highlight the importance of matching the cone angle with the specific geological conditions of the tunnel site.

### Engineering geological implications

The engineering geological characteristics of the surrounding rock significantly influence the effectiveness of blasting operations. In this study, the III-level surrounding rock exhibited higher integrity and lower fracture density compared to the IV-level surrounding rock. The higher stress concentration and crack propagation efficiency observed with the 65° cone angle in III-level rock suggest that this angle is better suited for more intact rock masses. Conversely, the 55° cone angle was more effective in the IV-level rock, which is characterized by higher joint development and weaker planes. This indicates that a smaller cone angle may be more appropriate for rock masses with higher fracture density, as it allows for more focused energy release and crack initiation.

The use of CCT also has implications for reducing the overall damage to the surrounding rock mass. By concentrating the explosive energy in a specific direction, the CCTs minimize the radial stress propagation, thereby reducing the extent of the crushed zone and the degree of crack propagation in non-cumulative directions. This is particularly important in tunnel construction, where maintaining the integrity of the surrounding rock is essential for long-term stability and safety.

### Practical application prospects

The practical application of CCTs in tunnel construction offers several advantages. The optimized cone angles identified in this study can be used to guide the design of blasting operations in similar geological conditions, thereby improving excavation efficiency and reducing costs. For instance, the reduction in the number of drill holes and the increase in contour hole spacing achieved through the use of CCTs can significantly decrease drilling time and explosive consumption. Additionally, the smoother tunnel wall surface resulting from the optimized blasting techniques can reduce the need for secondary support and improve the overall quality of the excavation profile.

Moreover, the CCTs can be easily integrated into existing blasting designs, making them a versatile tool for tunnel construction projects. The water wedge effect and energy storage capacity of water bags used in conjunction with CCTs further enhance the efficiency of explosive energy utilization and dust suppression, contributing to a more environmentally friendly and sustainable construction process.

### Limitations and future work

Despite the promising results obtained in this study, there are several limitations that need to be addressed in future research. First, the analysis of the potential mechanisms underlying the effectiveness of different cone angles was relatively superficial. A more in-depth investigation into the stress wave propagation, energy distribution, and fracture mechanics involved in CCT blasting is required to fully understand the underlying principles.

Second, the study focused primarily on III-level and IV-level surrounding rock conditions. The applicability of the findings to other rock mass classifications, such as II-level or V-level surrounding rock, remains uncertain. Future research should explore the performance of CCT in a broader range of geological conditions to establish more comprehensive guidelines for their use.

Finally, the long-term stability and potential secondary effects of CCT blasting on the surrounding rock mass require further investigation. Monitoring the post-blasting behavior of the rock mass over extended periods can provide valuable insights into the durability and safety of the tunnel structure.

In summary, while this study provides a solid foundation for the application of CCT in tunnel construction, further research is needed to optimize their design and expand their application to various geological settings. Future work should focus on a detailed analysis of the blasting mechanisms, validation in different rock mass classifications, and long-term monitoring of the tunnel structure.

## Conclusion

This study explores the optimization of C-type cumulative tube (CCT) cone angles for tunnel blasting in different surrounding rock conditions, revealing significant potential for improving excavation efficiency and minimizing damage to the surrounding rock mass. The key findings are summarized as follows:

Numerical simulations and field tests demonstrate that a 65° conical angle is optimal for III-level surrounding rock, reducing drill holes by 40%, over-excavation area by 22.7%, and maximum over-excavation value by 46.1%, while enhancing the half-hole retention rate by 24%. For IV-level rock, a 55° angle reduces drill holes by 43%, over-excavation area by 17.8%, maximum over-excavation value by 40%, and increases the half-hole retention rate by 17%.

The implementation of these optimized blasting schemes significantly improves tunneling efficiency by reducing drilling time and minimizing damage to the reserved rock mass. This ensures better blasting performance, contributing to higher precision and safety in tunnel construction.

The study innovation lies in its tailored approach to matching CCT conical angles with specific rock grades, offering practical guidance for optimizing blasting operations in diverse geological conditions. These findings have direct implications for reducing costs, improving excavation quality, and enhancing construction safety in modern tunnel engineering.

## Data Availability

The datasets generated during and/or analyzed during the current study are not publicly available but are available from the corresponding author on reasonable request.
